# Save the Forests

**Published:** 1875-08

**Authors:** 


### SAVE THE FORESTS.

The world, or some intelligent and respectable portion of it, is at last making up to the necessity of sparing the forests. The destructive process has heretofore been at work far more rapidly than the process of growth. Destruction has increased, as population and the wants of man have advanced, and vast territories have been denuded of their forest covering, and diverted to other uses. The result has been very serious.

If the forests of the world were wholly exterminated to-day, vast sections of the earth's surface would be uninhabitable. Experience proves that wherever forests are destroyed, there long and severe droughts occur, which dry up and destroy all vegetation. Accompanying these droughts are tremendous winds, which fill the air with dust and render it unfit to breathe. Then follow diseases of the lungs and air-passages, and mortality is greatly increased. All these results have followed the destruction of the forests in Austria and Hungary, where vast tracts of country have been entirely denuded by a species of worm which cuts off millions of great trees with the certainty and celerity of an epidemic. With the shelter thus lost, with the air rendered arid and parched, the injurious results which we have described are certain to follow. Human life is abbreviated. The soil loses its germinating power, and the land is rendered practically uninhabitable, except in the season of storms.

The change of climate thus induced, is almost marvellous. Time was when the climate of Pesth, Presburg and Vienna was delightful for human beings the whole year through. To-day, life is fairly intolerable there for fully nine months out of the twelve. At Rio de Janeiro, thunder storms and grateful showers, formerly of daily occurrence, are now rare; and the cause is clearly attributable to the destruction of the forests which surrounded the city. From this cause the yellow-fever has never been fully exterminated since the mountains were laid bare in 1852, notwithstanding the advance effected in modes of treatment. Realizing this, the authorities have directed that trees be thickly planted in every street; and this process is redeeming the land.

In Sweden the timber resources are immense; Lapland has never been surveyed, but is reckoned, with the northwestern provinces, to contain some 30,000,000 acres of forest. Unfortunately, the unceasing and enormous demands for wood, especially for charcoal, house building and lucifer matches, are telling rapidly on the power of the forests; this fact is of world-wide importance, for there is hardly a maritime country, except China and Japan, to which Swedish wood in some form does not find its way. At last, in 1874, a law was passed forbidding the felling of any trees less than seven inches in diameter.

In Switzerland, there is now a Sylvan Society, and great efforts are being made to induce people to re-plant cleared forest-land, so as to restore the climate to a healthfulness which it has partially lost at numerous points, as well as to prevent the damage which land-slides, floods and avalanches have of late years so frequently inflicted. This destruction of the Swiss forests has not been wholly wanton, since the leading industry of that country—wood-carving—has only been fostered by the use of vast quantities of timber. Cuba has abundant forests, her insurrectionary condition for many years having saved her wood from the axe.

Some curious experiments have been made in France to test how far the humidity of the atmosphere is affected by forests. Two sets of instruments for recording humidity were provided, one in a forest and the other in the open air, a short distance off, each set being placed about 50 feet from the ground. The records showed that during the first six months of 1874 more rain fell in the forest during each month than in the open field.

In no portion of the world are the results of irrigation as marked as on our Western Plains. To make the land inhabitable, it is simply necessary to inhabit it. By this we mean, that vegetable life makes animal life possible. Wherever the dust-heaps of the desert are irrigated and planted, man can live upon them. The very soil becomes changed. The evidence multiplies on every hand, that whenever the planet which we inhabit becomes treeless, all animal life will be extinguished. The lesson taught by this fact is that the man who robs the world of a tree, lessens by just so much the capacity of the world to support human life, and by so much abbreviates the life-supporting period of this planet; while he who plants and protects a tree, adds no insignificant portion to the well-being of humanity, and does something for the prolongation of the existing race of man.

